# A200 EXPOSURE TO CANNABIS SMOKE *IN UTERO* AFFECTS THE DEVELOPMENT OF THE ENDOCANNABINOID SYSTEM IN THE GASTROINTESTINAL TRACT

**DOI:** 10.1093/jcag/gwad061.200

**Published:** 2024-02-14

**Authors:** E Ratcliffe, M Sunil, T Podinic, J Kasinska, J Hirota, S Raha

**Affiliations:** McMaster University Faculty of Health Sciences, Hamilton, ON, Canada; McMaster University Faculty of Health Sciences, Hamilton, ON, Canada; McMaster University Faculty of Health Sciences, Hamilton, ON, Canada; McMaster University Faculty of Health Sciences, Hamilton, ON, Canada; McMaster University Faculty of Health Sciences, Hamilton, ON, Canada; McMaster University Faculty of Health Sciences, Hamilton, ON, Canada

## Abstract

**Background:**

Recreational use of cannabis has been legalized in Canada since 2018. Increasing numbers of women are using cannabis while pregnant, with reported rates ampersand:003E10%. There is a disconnect with societal perceptions, with up to one third of women believing that cannabis is safe for use in pregnancy, but with preclinical and clinical studies demonstrating negative neurodevelopmental outcomes. A key component of gut-brain axis regulation is found in the endocannabinoid system (ECS), of which receptors, ligands and enzymes are extensively distributed throughout the gastrointestinal (GI) tract and are actively involved in GI motility and sensation.

**Aims:**

We tested the hypothesis that exposure to cannabis smoke *in utero* could influence the expression of ECS components in the developing GI tract.

**Methods:**

We developed a “real world” cannabis smoke inhalation model in which CD-1 pregnant mouse dams were exposed daily to a delta-9-tetrahydrocannabinol (THC)-dominant strain of cannabis in a smoking chamber unit, from embryonic days 6 (E6) to E18. Control mice were placed in a restraining cage and exposed to room air for the equivalent time-period. Dams were sacrificed at E18, fetuses removed and the fetal GI tract (from stomach to distal colon) collected for RNA extraction. Key components of the ECS were investigated by developing a NanoString Custom CodeSet, including receptors (cannabinoid receptors 1 and 2; CB1 and CB2, transient receptor potential vanilloid type 1; TRVP1, GPR55) and enzymes (diacylglycerol lipase; DAGL, fatty acid amide hydrolase; FAAH, monoacylglycerol lipase; MAGL, *N*-acylphosphatidyletanolamine phospholipase D; NAPE-PLD). Samples (n=per litter; 7 cannabis-exposed and 8 control) were processed in the Farncombe Metagenomics Facility and NanoString data analyzed using nSolver Analysis Software.

**Results:**

We found a significant increase in the expression of ECS receptors in the cannabis-exposed compared to control GI tract at E18, including CB2 (pampersand:003C0.05), TRPV1 (pampersand:003C0.05) and GPR55 (pampersand:003C0.0005). Interestingly, no significant change in mRNA expression was detected for CB1. We also found a significant increase in mRNA encoding the full panel of ECS enzymes, including DAGL (pampersand:003C0.005), FAAH (pampersand:003C0.05), MAGL (pampersand:003C0.05) and NAPE-PLD (pampersand:003C0.005) in cannabis-exposed compared to control GI tract.

**Conclusions:**

In conclusion, we have found a significant increase in mRNA expression in a panel of key ECS receptors and enzymes in the GI tract at a point of maximal exposure to cannabis *in utero*. Future research will be needed to further characterize potential impacts on postnatal GI development and function.

**Funding Agencies:**

CIHRThe Azrieli Foundation

